# An Engineering Model of Magnetic Flux Density and Electromagnetic Force Density at the Structural Discontinuity within Transformer Cores

**DOI:** 10.3390/s22134869

**Published:** 2022-06-28

**Authors:** Lingzhi Li, Xuhao Du, Jie Pan, Xishan Jiang

**Affiliations:** 1 Key Laboratory of Intelligent Manufacturing Quality Big Data Tracing and Analysis of Zhejiang Province, China Jiliang University, Hangzhou 310018, China; 20a0102180@cjlu.edu.cn; 2 Department of Mechanical Engineering, The University of Western Australia, Perth, WA 6009, Australia; duxuhao88@gmail.com; 3 Department of Instrument Science and Engineering, Zhejiang University, Hangzhou 310027, China; jxishan@zju.edu.cn

**Keywords:** transformer core, air gap, electromagnetic force, magnetic flux density, analytical model

## Abstract

The structural discontinuities in the form of air gaps in transformer cores cause the concentration of electromagnetic force, which is an important source of transformer vibration and noise. In this paper, an engineering model of magnetic flux density and electromagnetic force density on transformer core discontinuities is analytically developed. Based on a reasonable structural simplification and assumptions, magnetic flux density and electromagnetic force density are deduced as explicit functions of the geometric, material, and electrical excitation characteristics of the gap region and the transformer core. The accuracy of the established model is validated by the finite element method (FEM) combined with a magnetic measurement experiment. According to this engineering model, the electromagnetic force density can be reduced by decreasing the gap ratio and increasing the gap thickness to a reasonable level. The outcome of this paper can help to understand the physical mechanism of the electromagnetic force generated by core air gap discontinuities, which is meaningful for noise control and the condition monitoring of transformers.

## 1. Introduction

One of the most important sources of transformer vibration and noise is the electromagnetic force in the core structure. A significant contributor to the electromagnetic force is the magnetic field discontinuity that results from air gaps at the overlapped region of the core due to the sudden change of permeability between the silicon steel sheets and air gaps [1,2,3]. Since electromagnetic force density is determined by the divergence of the magnetic field, it increases significantly at the discontinuity between the core materials and air gaps [4]. The previous study [5] indicated that the vibration due to the electromagnetic force and magnetostriction can be reduced in a specific frequency range with the proper design of the air gap size. Therefore, quantifying the effect of air gap discontinuities on the magnetic field and electromagnetic force is important for designing low-noise transformer cores.

The magnetic fields around the air gap discontinuities were mainly investigated by FEM and an experiment. Moses et al. measured the flux density distribution of the overlapped region. They found that the magnetic flux density in the inner side of the core was higher than the outer, and that the flux density was related to the core materials and transformer electrical excitation [6,7,8]. Ref. [9] calculated the localized magnetic flux density using FEM and a good agreement was obtained with the comparison of measured results. The investigated magnetic flux density was influenced by the geometry and magnetic material of the core. Shahrouzi established an FEM model in [10], and it was found that the computed flux density in the air gap and the gap bridge region were similar for both high permeability grain-oriented and low permeability non-oriented electrical steels. Configuration of the gaps had a bearing on the magneto resistance at the discontinuities. This result was further proved by [11], showing that flux transition in the overlapped region would be smoother with an improved stacking method. A more realistic scenario of magnetic flux transfer was included in this FEM simulation. Considering the technical challenges of the finite element meshing brought by air gaps, boundary conditions were added to thin, low permeability gaps for avoiding the real, thin air gaps in geometry, which implies that the reasonable modeling of air gaps is an important precondition for calculating the leakage magnetic field.

As a result of the discontinuous permeability and magnetic field, electromagnetic force and strain concentrate in overlapped air gap regions whose design thus plays a predominant role in noise generation [12]. In [13], the strain and displacement of a transformer core was measured, which depended on parameters such as material, stacking, clamping, magnetic induction, and rotational magnetization. The maximum magnetostriction was located on corners and T-joints as the structural discontinuities of the core. Weiser et al. [14] systematically studied the vibration of a transformer. They found that the discontinuities caused strain that was up to 10 ppm. Compared to the 0.3 ppm strain generated by magnetostriction, the electromagnetic force in the transformer core contributed more significantly to vibration. They fitted an empirical formula for boundary force density versus the step number and induced flux density to their FEM simulation results. However, this fitted formula cannot describe the effects of gap dimensions, core configuration, and material properties on the discontinuous boundary force. On the other hand, the electromagnetic force density in the core and its discontinuities can be calculated using the Maxwell stress tensor based on the virtual work principle. This method is widely used in calculating an inductor core’s vibration produced by its significant air gaps [15,16]. Nevertheless, for the study of electromagnetic force in transformer cores, the Maxwell stress tensor has been mainly used in FEM simulations, though rarely for explicit modeling owing to the difficulty of analyzing discontinuous magnetic fields [17].

It can be concluded from the above research that the magnetic field and electromagnetic force of the air gap discontinuities are directly related to the geometric, material, and electrical excitation characteristics of the gap regions and the transformer core. However, due to the tiny size and large quantity of air gaps, there exists no analytical model between air gaps and magnetic characteristics to intuitively describe magnetic fields and electromagnetic forces in the air gap discontinuities. This paper focuses on developing an engineering model to analytically predict the magnetic field and electromagnetic force density in the air gap discontinuities of transformer cores. In the subsequent model development, the overlapped structure of the air gaps is reasonably simplified to make analytical modeling feasible. The expressions regarding magnetic flux density and electromagnetic force density are deduced mathematically as functions of the characteristic parameters for air gaps and the transformer core. The simplified air gap setting is verified by comparing the FEM-simulated and experimentally measured magnetic flux density on air gap discontinuities. In order to validate the established model, the analytical results are compared with those of FEM under various air gap settings. The established engineering model can reveal the mathematical relationship of the magnetic field and electromagnetic force, along with the geometric and material parameters of the gap region. The deduced intuitive and explicit expressions are helpful to further understand the physical mechanism for leakage magnetic fields and electromagnetic force at the air gap discontinuities, which is an important source of transformer vibration and noise. Therefore, this understanding would aid engineering applications which require an estimation of electromagnetic force density, including the design of a low-noise transformer [18], as well as the fault diagnosis of transformers by indirectly monitoring the emitted vibration and extracting the vibration features [19].

## 2. Model Development

**Figure 1:**
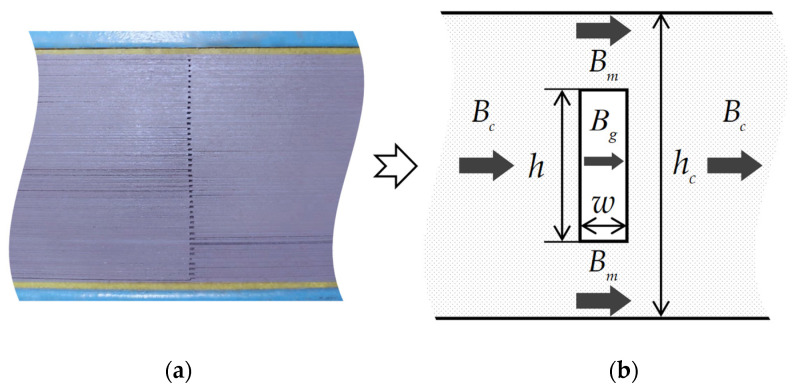
(**a**) Photo of the discontinuous air gap region within a transformer core in our laboratory; (**b**) the simplified air gap setting (top view).

The objectives of this paper involve the magnetic flux density and electromagnetic force in the discontinuous air gap regions of transformer cores, as shown in Figure 1. The gap region of cores is formed by the periodic overlapping of air and silicon steel sheets. Their permeabilities are thousands of times different, yet the gap size is commonly on the scale of millimeters, which means it is difficult to model every tiny air gap. Therefore, simplifying and modeling the air gap structure is the first step in this study. If all gaps can be equivalent to a single air gap in the middle of the core, as shown in Figure 1, then the difficulty of modeling can be greatly reduced. In this way, irregular overlapping errors in the core can also be avoided, thus improving the accuracy and operability of the developed engineering model. An experiment combined with FEM simulation will be conducted later in this paper to verify the equivalent air gap setting.

The magnetic flux flow over the core is shown in Figure 1, where $B_{g}$ is the magnetic flux density of the air gap, which is one of the modeling objects in this study and the other is the surface electromagnetic force density on the discontinuity air gap boundary; $B_{m}$ is the magnetic flux density of the core next to the air gap (defined as the side core); $B_{c}$ is the magnetic flux density of the core outside the air gap region; $h_{c}$ is the height of the core; and h and w are the height and width of the equivalent air gap, respectively. As a simplified engineering calculation model, $B_{c}$ is assumed to be uniform across the core, except in and around the air gap. This assumption will be investigated through FEM simulation. In general, the relative permeability of the core is thousands of times larger than that of the air, resulting in the great difference between $B_{m}$ and $B_{g}$. Due to the tiny dimension of the gap compared with the core, $B_{m}$ and $B_{g}$ are assumed to be uniform across the side core and the air gap, respectively.

$B_{g}$ is related to the gap size, the electromagnetic parameters and geometric size of the core, and the magnetic field outside the air gap region. Therefore, the magnetic flux density of the air gap $B_{g}$ can be divided into the induced magnetic flux $\Phi_{c}$ and transmission coefficient $b_{g}$, which are both related to the properties of the air gap, including the gap thickness w and air gap ratio $r_{g}=h/h_{c}$:

(1) $$B_{g}(r_{g},w)=b_{g}(r_{g},w)\Phi_{c}(r_{g},w).$$

Hence, the modeling of the magnetic flux density in the air gap is boiled down to the calculation of the transmission coefficient $b_{g}(r_{g},w)$ and the total flux $\Phi_{c}(r_{g},w)$. Then, the surface electromagnetic force density on the gap boundary can be calculated by the divergence of $B_{g}$.

### 2.1. The Induced Flux

**Figure 2:**
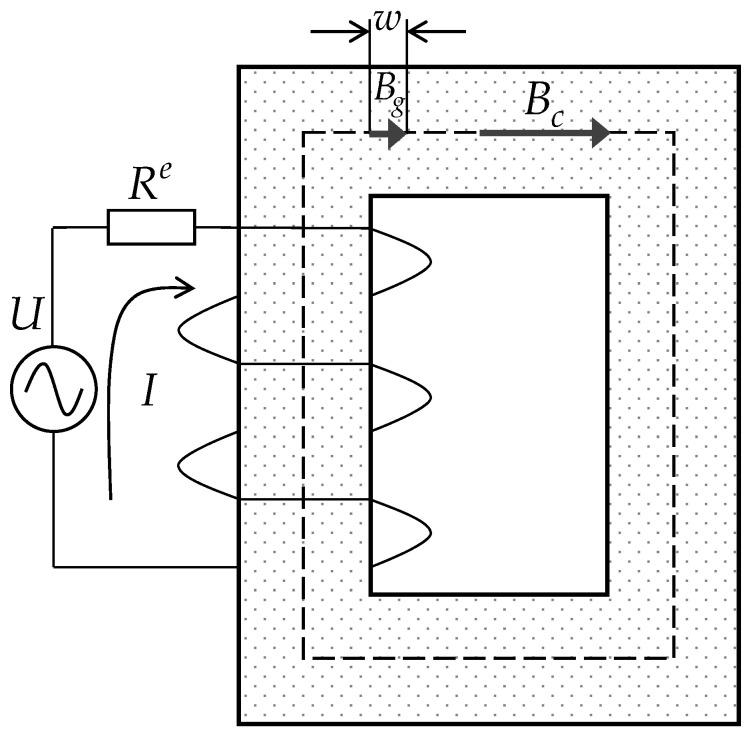
The electric circuit and magnetic flux flow over the core (front view).

The configuration of the electrical loop and magnetic loop of the single-phase transformer studied in this paper is shown in Figure 2. To highlight the effect of the air gap to the magnetic flux density and electromagnetic force, a single air gap is set to the transformer core in both the theoretical modeling and later simulation verification. U, I, and $R^{e}$ are the input voltage, current, and electrical resistance of the winding, respectively. The dotted line in the middle of the core represents the magnetic loop of the induced flux with the length of L.

According to the electrical circuit of the transformer,

(2) $$Ie^{-j\varpi t}R^{e}+N\frac{d\Phi_{c}e^{-j\varpi t}}{dt}=Ue^{-j\varpi t},$$

where ϖ is the excitation angular frequency and N is the number of the winding, with the assumption of $B_{c}$ being uniform in the core, $B_{c}$ can be calculated by the average value of $\Phi_{c}$ on the cross-section as $B_{c}=\Phi_{c}/S$, where S is the sectional area of the core. Therefore, the Ampere’s circuit law can be applied to the magnetic loop of the core in Figure 2 and the current in the winding can be related to the magnetic flux in the core as follows:

(3) $$\begin{array}{l} NI=\int_{0}^{w}\frac{B_{g}}{\mu_{0}}dl+\int_{w}^{L}\frac{B_{c}}{\mu_{0}\mu_{r}}dl\\ \;\;\;\;\;\;\;=\frac{B_{c}}{\mu_{0}\mu_{r}}l_{e} \end{array},$$

where $\mu_{0}$ and $\mu_{r}$ are the permeability of the vacuum and relative permeability of the core, respectively, and $l_{e}$ is the effective length of the magnetic path. 

Assuming there is no other magnetic flux leak from the core except the gap, the total flux in the core $\Phi_{c}$ can be divided into two parts: one through the core material $\Phi_{m}$ and the other through the air gap $\Phi_{g}$:

(4) $$\begin{array}{l} \Phi_{c}=\Phi_{m}+\Phi_{g}\\ B_{c}=(1-r_{g})B_{m}+r_{g}B_{g} \end{array}$$

The ratio of $B_{m}$ to $B_{g}$ is set to:

(5) $$K=\frac{B_{m}}{B_{g}}.$$

Substituting (4) and (5) into (3), the equivalent length of magnetic path is calculated as:

(6) $$l_{e}=L-w+\frac{w\mu_{r}}{K(1-r_{g})+r_{g}}l_{e}=L-w+\frac{w\mu_{r}}{K(1-r_{g})+r_{g}}.$$

Combining (2), (3) and (6), the total magnetic flux of the core can be described as a function of the excitation configuration (input voltage, excitation frequency, electrical resistance, and turn number of winding) and core configuration (core dimension, air gap dimension, and relative permeability of the core), as follows:

(7) $$\Phi_{c}=\frac{UNS}{-j\varpi N^{2}S+\frac{R^{e}(L-w)}{\mu_{r}\mu_{0}}+\frac{R^{e}w}{\mu_{0}K(1-r_{g})+\mu_{0}r_{g}}}.$$

Combining (4) and (5), $B_{g}$ can be expressed by $\Phi_{c}$ as

(8) $$B_{g}=\frac{1}{r_{g}+K(1-r_{g})}\Phi_{c}.$$

Equation (8) is the further description of (1). Thus, the transmission coefficient $b_{g}$ is determined by:

(9) $$b_{g}=\frac{1}{r_{g}+K(1-r_{g})}.$$

It can be observed from (9) that $b_{g}$, $B_{g}$, and $\Phi_{c}$ can be calculated as long as the relationship between K and the core configuration is obtained. Therefore, the ratio between the magnetic flux density of the side core $B_{m}$ and the magnetic flux density of the air gap $B_{g}$ will be deduced in the following subsection.

### 2.2. Magnetic Flux Density in the Air Gap

**Figure 3:**
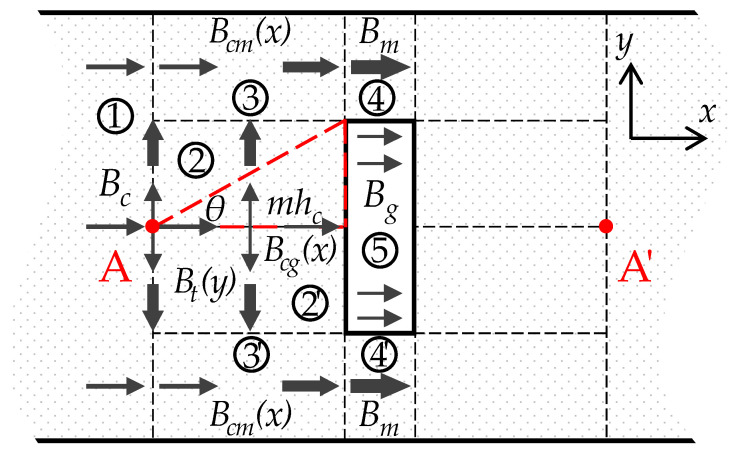
The magnetic flux density distribution around the air gap region.

The component of the magnetic flux density near the air gap region is represented in Figure 3. The air gap region from A to A′ is the main focus of this subsection. In the coordinate, x=0 is the center line of the yoke and y=0 is the left boundary of the gap (the one closer to the joint). The different arrow widths indicate the different amplitudes of the magnetic flux density, and the different numbers denote the regions with different magnetic flux properties. The magnetic flux characteristics are symmetrical along A-A′. Region No. 2′, No. 3′, and No. 4′ are symmetric with Region No. 2, No. 3, and No. 4, respectively.

Due to the significant difference in the relative permeability between the air and the core material, most flux is redirected through the side core. According to the previous FEM study [20,21], the change in direction of the magnetic flux can be considered to start at a certain point (the turning point A in Figure 3) close to the air gap. It is obvious that with a larger $h_{c}$, this turning point occurs earlier. Thus, a coefficient m is introduced so that the distance from the turning point to the air gap can be expressed as $mh_{c}$, whose included angle with the edge of the gap is θ. It may be noted that this starting point angle is approximately a constant according to the reverse calculation from the FEM simulation results and (18). This will be discussed in Section 4:

(10) $$m=\frac{r_{g}}{2\tan\theta }.$$

The transverse flux that changes direction from *x* direction to *y* direction is defined as $\Phi_{t}$. Point A represents the location where $\Phi_{t}$ starts to appear, and the flux approaches $\Phi_{t}(r_{g}h_{c}/2)$ at the boundary of region No. 2 and No. 3. Therefore, the magnetic flux density of *y* direction in region No. 2, $B_{t}(y)$, increases from 0 at point A to $B_{t0}$ at the boundary between region No. 2 and No. 3. As a result, the flux densities in the *x* direction in regions No. 2 and 3, which are expressed as $B_{cg}(x)$ and $B_{cm}(x)$, respectively, gradually decrease and increase from point A to the discontinuity boundary, respectively. Analogous to Kirchhoff’s current law in an electrical circuit, (11) can be obtained in region No. 3 from Gauss’s law [22]:

(11) $$\frac{1-r_{g}}{2}\Phi_{c}+\Phi_{t}(r_{g}h_{c}/2)=\Phi_{m}.$$

Equation (11) indicates that the magnetic flux flowing into and out of region No. 3 is equal. The summation of the part of the core magnetic flux in region No. 1 that passes into region No. 3 and the magnetic flux that turns around from region No. 2 to region No. 3 is equal to the magnetic flux that passes through region No. 3 to No. 4.

**Figure 4:**
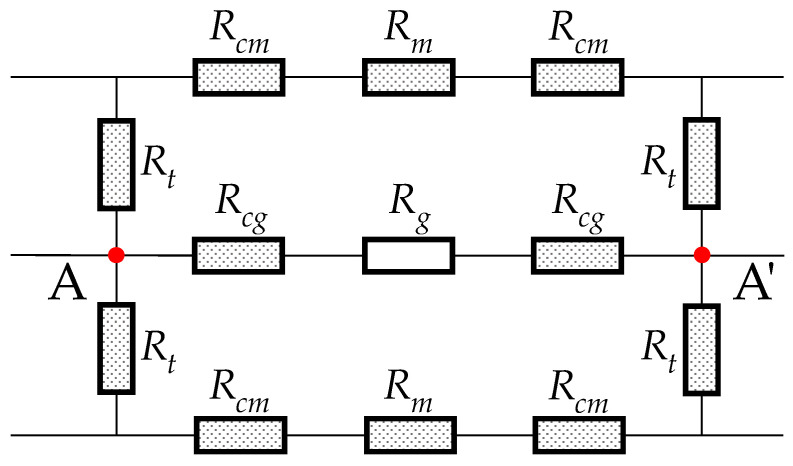
The magnetic circuit of the gap region.

There are two types of magnetic paths from point A to A′, as shown in Figure 4. According to Hopkinson’s law of the magnetic path, the drop of magnetomotive force caused by passing the magnetic reluctance though two different paths should be equal. The potential drop through the first path includes passing the reluctances $R_{t}$, $R_{cm}$, and $R_{m}$, which is represented by the left term in (12). The one through the second path includes passing the reluctances $R_{cg}$ and $R_{g}$, which is represented by the right-hand term in [23]:

(12) $$2\int_{0}^{r_{g}h_{c}/2}\Phi_{t}(y)dR_{t}+2\int_{-mh_{c}}^{0}\Phi_{cm}(x)dR_{cm}+R_{m}\Phi_{m}=2\int_{-mh_{c}}^{0}\Phi_{cg}(x)dR_{cg}+R_{g}\Phi_{g},$$

where $R=l/S\mu_{0}\mu_{r}$ is the magnetic reluctance. To the first order approximation, $\Phi_{t}(y)$ is assumed to change linearly along the *y* direction. Subsequently, magnetic flux densities at regions No. 2 and 3 can be expressed by (13) to (15):

(13) $$B_{t}(y)=\frac{B_{t0}}{r_{g}h_{c}/2}y,$$

(14) $$B_{cm}(x)=\frac{B_{m}-B_{c}}{mh_{c}}x+B_{m},$$

(15) $$B_{cg}(x)=\frac{B_{g}-B_{c}}{mh_{c}}x+B_{g},$$

where $x\in [-mh_{c},0],\;\;y\in [0,r_{g}h_{c}/2]$. Combining (4) and (11) to (15), the ratio *K* is obtained as:

(16) $$K=1+\frac{4m(\mu_{r}-1)w}{h_{c}r_{g}{}^{2}(1-r_{g})+4m(w+mh_{c})}.$$

If $\mu_{r}=1$, for the air gap, K=1 so that $B_{m}=B_{c}$. With gap thickness increasing, the enlarged reluctance of the air gap forces more flux to pass through the side core, which leads to an increase in *K*. Substituting (16) into (7) to (9), magnetic flux density of the side core $B_{m}$ and the magnetic flux density of the air gap $B_{g}$ are calculated by:

(17) $$B_{m}=\frac{UN}{-j\varpi N^{2}S+\frac{R^{e}(L-w)}{\mu_{r}\mu_{0}}+\frac{R^{e}w}{\mu_{0}K(1-r_{g})+\mu_{0}r_{g}}}\frac{K}{r_{g}+K(1-r_{g})},$$

(18) $$B_{g}=\frac{UN}{-j\varpi N^{2}S+\frac{R^{e}(L-w)}{\mu_{r}\mu_{0}}+\frac{R^{e}w}{\mu_{0}K(1-r_{g})+\mu_{0}r_{g}}}\frac{1}{r_{g}+K(1-r_{g})}.$$

From (17) and (18), it is clear that increases in input voltage and winding turns cause larger magnetic flux density in both the side core and air gap, which matches the physical understanding of the transformer system. Based on these two deduced expressions of $B_{m}$ and $B_{g}$, electromagnetic force density in the air gap region can be analyzed in the next subsection.

### 2.3. Electromagnetic Force Density

The electromagnetic force density is determined by the divergence of the Maxwell stress tensor [4]:

(19) $$f_{v}^{c}=\nabla \cdot T^{c},$$

where $T^{c}$ is the Maxwell stress tensor defined by:

(20) $$T^{c}=BH-\frac{\mu_{0}}{2}H^{2}I,$$

where B and H are magnetic flux density and magnetic intensity, respectively, $\mu_{0}$ is the permeability in the vacuum, and I is the identity matrix. The surface electromagnetic force density on the gap boundary is expressed as:

(21) $$f_{s}^{cd}=n\cdot (T_{e}^{c}-T_{i}^{c}),$$

where n is the outward normal unit vector of the air gap boundary and $T_{e}^{c}$ and $T_{i}^{c}$ are the Maxwell stress tensors of the external side (air side) and internal side (core side) of the boundary surface, respectively. 

By ignoring the hysteresis effect inside the core, the relationship between the magnetic flux density B and the magnetic intensity H is:

(22) $$H=\frac{1}{\mu_{0}\mu_{r}}B.$$

According to Maxwell’s equation, the normal components of the magnetic flux density for the core and air are continuous at the contact boundary. Thus, the relationship between the core magnetic flux density on the gap edge interface $B_{cg0}$ and the air gap magnetic flux density $B_{g}$ can be described by:

(23) $$n\cdot B_{cg0}=n\cdot B_{g}.$$

Combining (19) to (23), the surface electromagnetic force density in (21) can be further expressed as:

(24) $$f_{s}^{cd}=n\cdot [\frac{B_{g}\cdot B_{g}^{T}}{\mu_{0}}(1-\frac{1}{\mu_{r}})-\frac{B_{g}{}^{2}}{2\mu_{0}}(1-\frac{1}{\mu_{r}{}^{2}})I].$$

Since the relative permeability $\mu_{r}$ is usually much larger than 1, the term $1/\mu_{r}$ in (24) is negligible. Subsequently, the surface force density can be expressed as a function of the magnetic flux density $B_{g}$ at the boundary of the discontinuity, as follows:

(25) $$f_{s}^{cd}=\frac{B_{g}{}^{2}}{2\mu_{0}}.$$

Substituting (18) into (25), the surface electromagnetic force density on the discontinuity gap boundary is finally expressed by:

(26) $$f_{s}^{cd}={\left[\frac{UN}{-j\varpi N^{2}S+\frac{R^{e}(L-w)}{\mu_{r}\mu_{0}}+\frac{R^{e}w}{\mu_{0}K(1-r_{g})+\mu_{0}r_{g}}}\frac{1}{r_{g}+K(1-r_{g})}\right]}^{2}\frac{1}{2\mu_{0}}$$

It can be seen from (26) that most of the parameters such as the air gap ratio, permeability, and gap thickness appear both in the denominator and numerator, and thus it is difficult to infer directly how the air gap setting influences the boundary force density. The numerical and graphical approaches will be used to infer the influences.

## 3. Experiment and FEM Simulation

**Figure 5:**
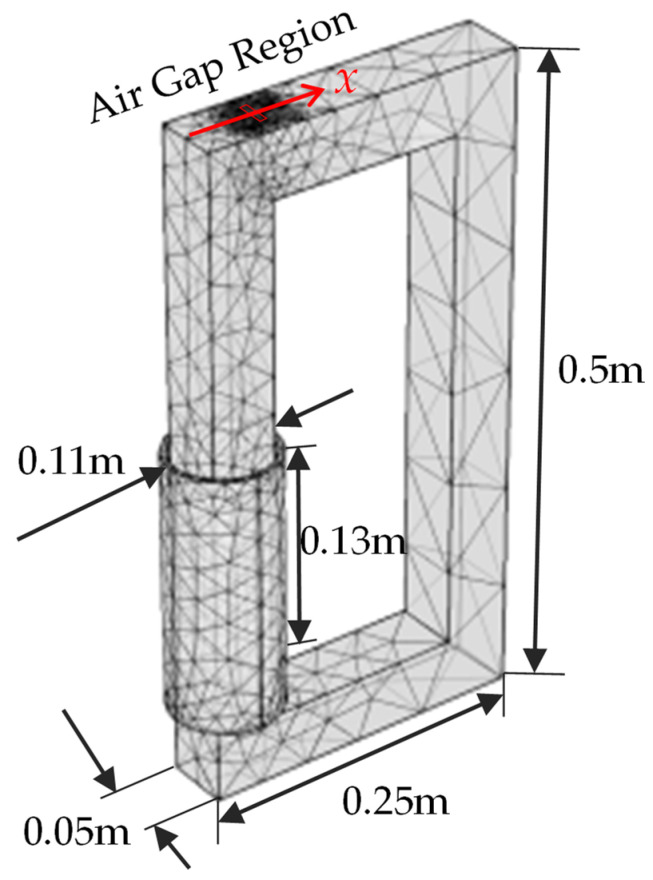
The configuration and FEM meshing of the transformer.

In order to verify the simplified equivalent air gap setting, an actual single-phase transformer core in our laboratory is used for the magnetic measurement experiment, whose configuration and FEM meshing of the transformer are shown in Figure 5. The number of turns in the winding *N* is 200 and the winding resistance $R^{e}$ is 27.9 Ω. The transformer is a no-load and the input voltage U is 200 V with an excitation frequency of 50 Hz. Due to the difficulty of directly measuring the magnetic field and force inside the tiny air gap [24], leakage magnetic flux density over the yoke surface is measured with a hall sensor (accuracy: 10  μT, size: 4 mm×5 mm×1.5 mm). The core structure is symmetrical along the *x*-axis and the air gaps are periodically distributed along the *y* axis by overlapped silicon steel sheets. Therefore, the measurement is conducted on the yoke center line (i.e., the *x*-axis), where −50 mm≤x≤50 mm, and the air gaps are located on x=0 mm. Twenty-one measuring points are evenly arranged within 100 mm, and thus the spacing between the two measuring points is 5 mm. The hall senor is successively attached on each measuring point of the yoke surface to measure the magnetic flux density along the *x* direction, which is the direction of the modeled $B_{g}$. For further comparison, the hall sensor is then placed 2 mm above the original measuring point to measure the leakage magnetic flux density from the yoke surface, which can be compared with the surface measurement. The results of two measurement experiments will be shown in the next section.

Based on the FEM simulation software COMSOL Multiphysics (v.5.4, COMSOL Co., Ltd., Stockholm, Sweden), the electromagnetic field module and circuit module are included in the simulation. The configuration parameters, such as dimensions, input voltage, and winding turns, are consistent with the actual transformer. The air gap ratio and thickness are 0.5 and 2.2 mm, respectively. For comparison with the measurement experiment, the leakage magnetic flux density on the yoke surface center line and 2 mm off the line are simulated by FEM, and the results are shown in the next section.

FEM is also used to gain confidence in the analytical derivation. Different dimensions of the air gap are used as the gap ratio $r_{g}$ changes from 0.1 to 0.9, stepped by 0.1, and the air gap thickness w changes from 2 mm to 18 mm, stepped by 2 mm. The gap ratio is normally no larger than 0.5, while, technically, if one does the overlap insertion randomly, the ratio can be higher than 0.5. To maintain this possibility, an $r_{g}$ larger than 0.5 is also calculated and simulated in this study. Five different relative permeabilities ($\mu_{r}$) are used, including 1000, 2500, 5000, 10,000, and 20,000, which cover the range of the relative permeability of common silicon steel materials. Thus, a total of 405 different conditions are simulated and analyzed.

## 4. Results and Discussion

### 4.1. Verification of Model Assumptions

**Figure 6:**
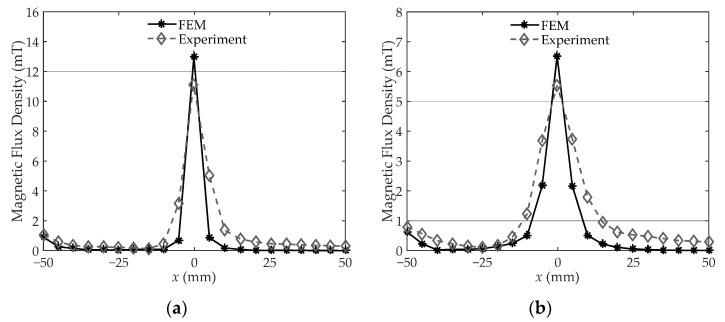
The leakage magnetic flux density (**a**) on the yoke surface center line; (**b**) 2 mm off the line.

The experiment is implemented to justify the equivalent air gap and FEM simulation. The leakage magnetic flux density on the yoke surface center line and 2 mm off the line are investigated and shown in Figure 6a,b, respectively. The FEM results are close to the actual measured magnetic flux density in the two measuring lines, which validates that the magnetic characteristics of the overlapped assembly air gaps can be well characterized by the simplified equivalent air gap. Located at *x* = 50 mm, the air gap magnetic flux density is significantly higher than in other regions, and it appears in a single peak distribution. The FEM-simulated results are slightly lower than the measured results, which may be caused by the more concentrated air gap configuration. 

For the region 5 mm away from the air gap, the magnetic field plunges to less than 1 mT, which indicates that the magnetic flux density changes drastically around the gap. Besides, simulated magnetic flux density is slightly lower than that of the experiment because the actual core inevitably leaks magnetic flux from the laminated silicon steel sheets to air in the area outside the gap region, which increase the measured magnetic flux. Comparing Figure 6a,b, the leakage magnetic field is almost reduced by half when the measuring sensor moves outward 2 mm, which further proves the great decrease of the air gap magnetic field from the normal direction of the core surface.

The FEM configuration is basically consistent with the experiment, except for the equivalent air gap setting. Therefore, the experiment results over the air gap region are more accurate than the FEM results based on equivalent configuration. On the other hand, for the region outside the air gap, the FEM results are supposed to be more accurate than the experiment. The inevitable leakage magnetic flux from the actual irregular laminated silicon steel sheets increases the magnetic flux in this region, which makes the measurement difficult, and the hall sensor may measure some magnetic flux produced by irregular laminated sheets. Based on the above observation and discussion, the magnetic peak over the gap and the sharp magnetic attenuation away from the gap both prove that the leakage magnetic flux can be treated as originating from one concentrated air gap, i.e., the simplified air gap setting is reasonable for the model development.

**Figure 7:**
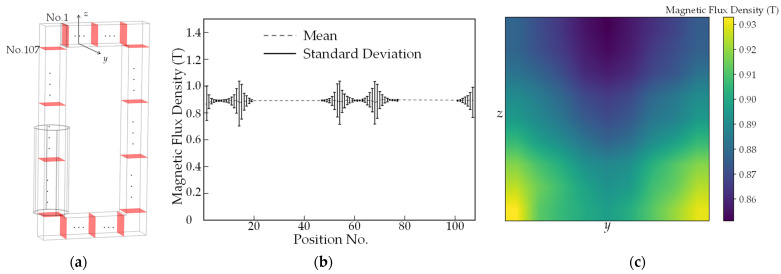
(**a**) The 107 selected equidistant slices of the core; (**b**) The FEM-simulated area-averaged magnetic flux density of core cross-sections over the cycle with the surface standard deviation; (**c**) The magnetic flux density distribution on slice No. 2 as a transient stage near the corner.

To support the assumption of uniformity used in (9), 107 equidistant slices of the core are selected, as shown in Figure 7a, whose magnetic flux densities along the rolling direction $B_{c}$ are simulated using FEM. The results of the area-averaged $B_{c}$ with the surface standard deviation are shown in Figure 7b. The standard deviation of the magnetic flux density changes sharply over four core corners where air gaps located. Nevertheless, the area-averaged $B_{c}$ is stable, with only a 0.5% coefficient of variation and 2.2% averaged surface standard deviation, while the variations of $B_{c}$ under other air gap settings over the core give similar results. The standard deviation of each slice shows some variation at the corner positions due to the different lengths of the magnetic paths. The largest relative difference appears on the slice at the corner and air gap. The magnetic flux density distribution on the slice of No. 2 as a transient stage near the corner is shown in Figure 7c. It can be seen that magnetic flux density of the inner side is higher (the bottom part of Figure 7c) than the outer side (the upper part of Figure 7c). Along the *y*-axis, the central part is smaller than the two sides because of the different lengths of magnetic paths and the effect of the air gap. The relative difference between the maximum and minimum values of this corner slice is 7.8%. The variations of $B_{c}$ and relative difference of the corner slices are small enough to warrant the assumption that the area-averaged $B_{c}$ is reasonably uniform across the whole core, and thus proves the rationality of adopting Ampere’s circuit law in (12). Compared with the whole core, the geometry size of the air gap area is small. In this case, the above analysis regarding $B_{c}$ also provides confidence to assume $B_{g}$ and $B_{m}$ are uniform in the previous modeling.

Equation (16) shows the relationship between the ratio *K* and the starting point angles. From [5,16], the angle can be reversely calculated based on the same equation from the FEM simulation results, which yields a mean value of $\theta =81^{\circ }$ with a standard deviation 1.8° from all 405 air gap settings. The coefficient of variation is 2.3%, which is small enough to support the stability of the starting angle. Therefore, the distance between starting point A and the discontinuity boundary can be expressed as $r{}_{g}h_{c}/\left(2\tan\theta \right)$, which indicates that the larger the gap ratio, the earlier the flux starts to change direction. The large θ angle is very close to 90°, that is, the flux changes direction when it is very close to the discontinuity. This can be confirmed by Figure 7. When magnetic flux approaches the air gap, part of the flux starts to turn from a rolling direction to a transverse direction. The change of magnetic flux direction increases the nonuniform magnetic distribution of the core cross-section, and then causes the deviation of the area-averaged magnetic flux density, which concentrates on the air gap region of the core.

### 4.2. Comparison between the Engineering Model and FEM Simulation Results

**Figure 8:**
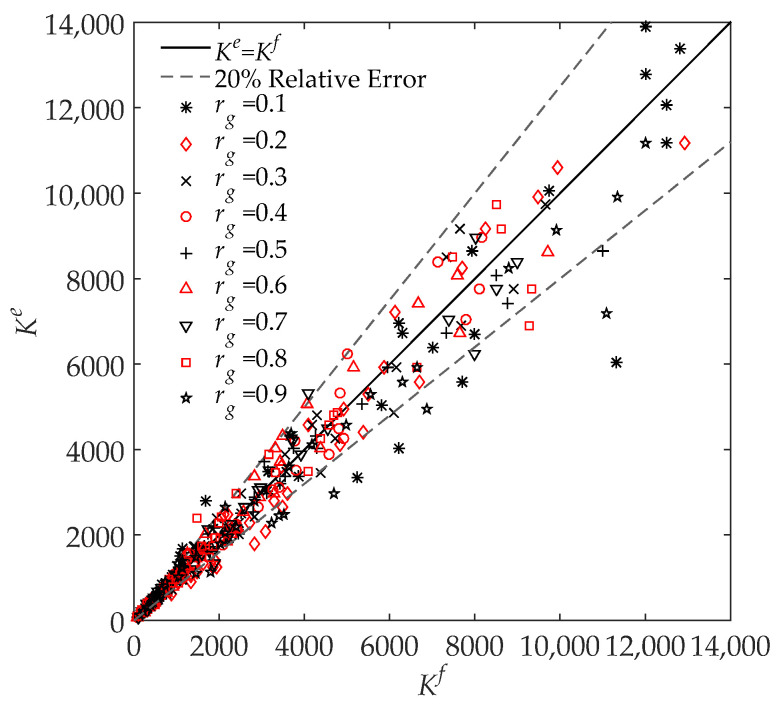
FEM-simulated $K^{f}$ and analytical $K^{e}$ under all 405 air gap settings. If the FEM result is the same as the analytical result, the point should fall on the black perfect line $K^{e}=K^{f}$.

The results from the FEM and the engineering model are presented and discussed in this section. The ratio of the simulated $B_{m}$ to $B_{g}$, defined as $K^{f}$, can be obtained by changing $r_{g}$, $\mu_{r}$, and w in the FEM simulation. Meanwhile, the analytical $K^{e}$ can be calculated by (16). Figure 8 compares both the $K^{f}$ and $K^{e}$ results under all different gap thicknesses, gap ratios, and relative permeabilities, where the *x*-axis and *y*-axis represent the FEM $K^{f}$ and the analytical $K^{e}$, respectively. If the FEM result is the same as the analytical result, the point should fall on the perfect curve (black line), which is $K^{e}=K^{f}$. Both the grey lines indicate the 20% relative error. 

From the comparison between the FEM and analytical results, it is clear that most of the relative errors are smaller than 20%, with an average of 12.7%. The small relative error indicates that the analytical model developed in this paper can reasonably predict the relationship between $B_{m}$ and $B_{g}$. There are some poor prediction results for $K^{e}$, which are located below the lower grey line. This may be caused by the assumption that $\Phi_{t}$ changes linearly along the *y* direction in region No. 2. The overall trend of this simplification is true, where $\Phi_{t}$ should increase monotonically along the *y* direction. However, this increase may not be linear, which leads to the small calculation error of the developed model. Nevertheless, the analytical model based on this linear simplification has achieved an accurate prediction with small error, and thus further improvement is an area for future work. From Figure 8, it can be observed that $B_{m}$ is approximately thousands of times that of $B_{g}$, which is consistent with the relevant research [14].

**Figure 9:**
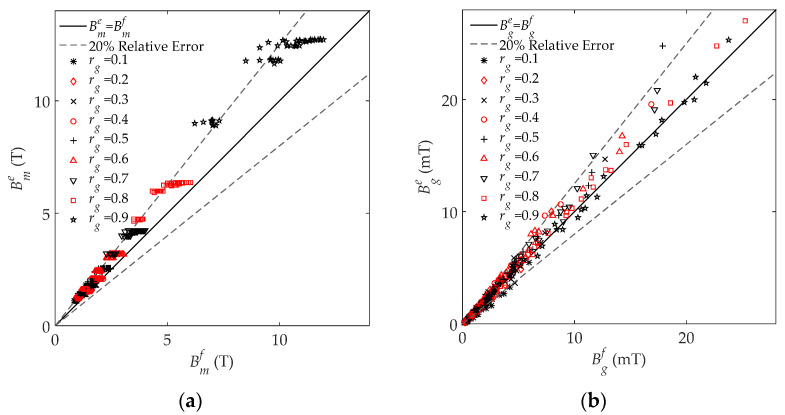
(**a**) FEM-simulated $B_{m}^{f}$ and analytical $B_{m}^{e}$ under all 405 air gap settings. If the FEM result is the same as the analytical result, the point should fall on the black perfect line $B_{m}^{e}=B_{m}^{f}$; (**b**) FEM-simulated $B_{g}^{f}$ and analytical $B_{g}^{e}$ under all 405 air gap settings. If the FEM result is the same as the analytical result, the point should fall on the black perfect line $B_{g}^{e}=B_{g}^{f}$.

Finally, according to (17) and (18), similar comparisons between the FEM and analytical results of both $B_{m}$ and $B_{g}$ are presented in Figure 9a,b, with average relative errors of 10.1% and 12.6%, respectively. There are few of the calculated results located outside the 20% relative error line. From Figure 9a, most of the $B_{m}$ that are out of the relative error lines correspond to $r_{g}=0.9$, which is an extreme air gap width (generally $r_{g}\leq 0.5$). When the gap width is too large, the nonlinearity of $B_{t}$ in the *y* direction also increases and leads to the modeling error of $B_{m}$. Similarly, the assumptions of the developed model lead to the deviation of some $B_{g}$, as shown in Figure 9b. The finite element mesh generation criteria also have a certain impact on the FEM simulation and causes the difference between the FEM and analytical results.

### 4.3. Effect of the Air Gap on the Magnetic Field

**Figure 10:**
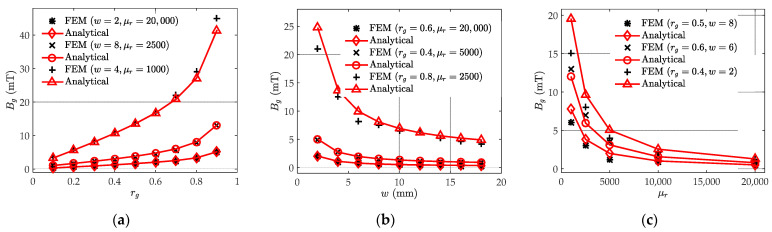
Comparison between the FEM simulation (black markers) and analytical (red curves) leakage magnetic flux density $B_{g}$ with the change of (**a**) air gap ratio $r_{g}$; (**b**) air gap thickness w; (**c**) relative permeability $\mu_{r}$.

The detailed comparison under different air gap settings versus gap ratio, width, and relative permeability are shown in Table 1. For the three variables $r_{g}$, w, and $\mu_{r}$ in the developed model, three cases are set respectively to study the influence of each variable on the magnetic flux density in the gap. As shown in Figure 10, the analytical results (red curves) of the engineering model are compared with those of the FEM (black markers).

As seen in Figure 10, the FEM and analytical results match well under varied air gap settings and relative permeabilities. As shown in Figure 10a, with an increasing gap ratio $r_{g}$, $B_{g}$ increases significantly because of the reduced reluctance ratio between the air gap and the side core $R_{g}/R_{m}=(1-r_{g})/r_{g}\mu_{r}$ (according to the definition of magnetic reluctance). On the other hand, with the increasing gap thickness w, $B_{g}$ decreases significantly, as shown in Figure 10b. This is because with a larger travel distance, $B_{g}$ requires a much larger magnetic potential. Therefore, more magnetic flux turns to pass through the side core. This phenomenon is similar to fluid flow through a pipe. As flow moves along the pipe, a laminar flow is established and provides a stronger resistance, which results in a small gap ratio and a reduced flow passing through. After travelling a certain distance, the laminar flow enters the fully developed region and the resistance and velocity become stable [25]. As seen in Figure 10c, a smaller relative permeability causes a larger $B_{g}$. The increase of $B_{g}$ is caused by the reduced difference between the core material permeabilities and air permeabilities. In this case, the magneto resistance difference is also reduced and more magnetic flux flows into the air gap, which finally results in the increase of magnetic flux density in the gap. 

### 4.4. Effect of the Air Gap on Electromagnetic Force Density

**Figure 11:**
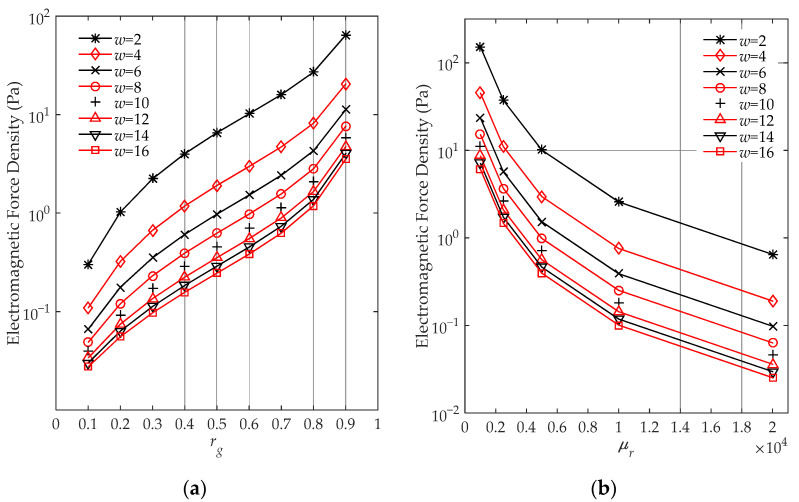
The electromagnetic force density against different air gap ratios and air gap thicknesses when (**a**) $\mu_{r}=8000$; (**b**) $r_{g}=0.4$.

To investigate the influence of air gap on the electromagnetic force density at the discontinuity boundary, $f_{s}^{cd}$ are calculated under varied parameters of air gap based on (26). Figure 11a presents the force density under a constant relative permeability $\mu_{r}=8000$ and different air gap dimensions, including the different gap thicknesses w of 2 to 16 mm, stepped by 2 mm, and gap ratios $r_{g}$ of 0.1 to 0.9. Similarly, under the same gap thickness w variation setting, Figure 11b shows $f_{s}^{cd}$ under a constant gap ratio $r_{g}=0.4$ and different relative permeability $\mu_{r}$ of 1000 to 20,000. The force density in the *y*-axis is presented in log-scale in Figure 11a,b.

The calculated force density level matches that of previous studies [5,15]. From Figure 11a, it is clear that under the same gap ratio, the smaller the gap thickness, the larger the force will be. A smaller gap thickness causes a stronger magnetic flux leakage owing to the lower magnetic reluctance, which results in a larger force density. On the other hand, with a larger air gap ratio, the discontinuity force density increases exponentially, which gives us theoretical evidence that the multistep layer joint introduces less boundary force than the single-step assembly joint. Following this result, when designing a low-noise transformer, one should use larger numbers of step layers at the joint connection and compress core lamination in the gap region. This discovery is consistent with the previous experiment by Weiser et al. [14] in which the shown surface vibration and noise will be significantly reduced when $r_{g}$ decreases.

In addition, from Figure 11b, a smaller relative permeability causes a larger force. This is because the relative permeability difference between the core and the air reduces the flux that passes through the air gap. Due to the significant change in magnetic field strength at the discontinuity of the core (approximately $\mu_{r}$ times compared to other locations of the core; see (22) and (23)), the surface electromagnetic force density $f_{s}^{cd}$ may be significantly larger as a result of the gap setting and excitation configuration in (26). As shown in both Figure 11a,b, the engineering model indicates that the force density reduces when the gap thickness increases. However, the large gap thickness will introduce a rotation magnetization that makes the core magnetic flux non-uniform [26], which reduces the transformer efficiency in terms of reduction of magnetic flux for a given input [27]. Therefore, it is necessary to consider the effects of air gaps on both force density and efficiency when designing a transformer core.

### 4.5. Discussion about the Assumptions and Applications of the Engineering Model

#### 4.5.1. Discussion about the Assumptions of the Engineering Model

It can be seen from the above results and discussion that the proposed engineering model can effectively calculate the magnetic field and electromagnetic force on the transformer core discontinuity. However, to a certain degree, the assumptions of the model influence the accuracy in predicting magnetic characteristics. Three assumptions mainly used in developing the engineering model and the caused accuracy issues are explained and discussed as follows:

**Assumption** **1.** *All air gaps are equivalent to a single air gap in the middle of the core. As the basic equivalent setting of the study, this assumption is proposed at the beginning of modeling to simplify the complex overlapping structure of air gaps and ensure modeling operability. A magnetic experiment combined with FEM justifies the equivalent setting, as shown in*Figure 6. *With the more concentrated gap setting, this assumption leads to the larger calculated magnetic flux density of air gap region. The small difference between the experiment and the FEM indicates that this assumption is reasonable.*

**Assumption** **2.** $B_{c}$*is uniform across the core, except in and around the air gap.*$B_{m}$*and*$B_{g}$*are uniform across the side core and the air gap, respectively. This assumption is proposed before the model deduction and immediately following Assumption 1. The assumption of uniformity reduces the engineering modeling difficulty, whose reliability is investigated the FEM simulation, as shown in*Figure 8. *The small coefficient of variation and small averaged surface standard deviation provide confidence to assume*$B_{c}$*,*$B_{g}$*, and*$B_{m}$*are uniform in the modeling. This assumption may lead to the calculated magnetic flux density and electromagnetic force density being smaller than the actual because some magnetic changes are ignored, and these changes can give rise to more leakage magnetic flux and electromagnetic force.*

**Assumption** **3.** $\Phi_{t}$*changes linearly along the y direction. This assumption is used for the expression of*$\Phi_{t}$*on deducing Kirchhoff’s current law and Hopkinson’s law. This assumption simplifies the derivation procedure and makes the engineering model clear. Further, the overall trend is a monotonic increase, but not necessarily linear, which results in the calculation errors of*$B_{g}$*and*$B_{m}$*. The reasonability of this assumption is verified by the comparison with FEM and the results are shown in*Figure 9*and*Figure 10. *Investigations about*$\Phi_{t}$*will be the subject of further work in the future.*

#### 4.5.2. Discussion about Applications of the Engineering Model

Low-noise core design and condition monitoring for transformers are the two main applications of the proposed model. A detailed description and further investigations about these two applications are discussed as follows:

1. Application 1: The electromagnetic force generated by air gap discontinuities is a significant source of the noise of a laminated core. Therefore, proper design of the air gap geometry and selection of the core material can control the noise produced by electromagnetic force. According to the model established in this research, a smaller air gap ratio, larger gap thickness, and larger core permeability all contribute to a lower leakage magnetic flux and electromagnetic force. This indicates that engineers can reduce core noise by using silicon steel sheets with better magnetic permeability, increasing the gap thickness and larger numbers of step layers at the gap region. However, excessive air gap thickness increases the core loss of the transformers. The design of cores must give consideration to both noise and efficiency, whose balance is the subject of further investigation and application of the developed engineering model.
2. Application 2: The vibration on transformer tanks contains abundant information about the internal condition of the transformers. Analysis of the vibration, combined with vibration mechanism modeling, can monitor transformers in operation. The vibration produced by the electromagnetic force in the core discontinuity air gap region contributes a lot to vibration on the transformer tanks. Once the core structure looseness or insulation damage appears inside the transformer, the magnetic and force characteristics of the gap region will change accordingly. The mechanism of this change can be deduced from the proposed model because looseness and insulation damage can affect the gap size and core permeability. Therefore, the developed engineering model can help in monitoring transformer conditions. The mechanism of vibration produced by the electromagnetic force needs further research. Fault characteristics extraction based on the vibration mechanism is also a key step in using the engineering model for transformer monitoring.

## 5. Conclusions

In this paper, an engineering model of magnetic flux density and electromagnetic force density on transformer core discontinuity is established analytically. Based on Ampere’s circuit law, Gauss’s law, and Hopkinson’s magnetic path law, the magnetic flux density and electromagnetic force density are expressed as functions of the air gap ratio, gap thickness, relative permeability, core configuration, and excitation setting. FEM is used to compare the corresponding calculated magnetic field under various air gap settings. A good agreement between the FEM result and the analytical model is obtained, which indicates the accuracy of the developed model. From the engineering model, it is clear that the increased air gap ratio increases the magnetic flux density and electromagnetic force density. Conversely, increasing the air gap thickness reduces the leakage magnetic field and force density. Decreased relative permeability results in a larger force density. According to this model, the electromagnetic force density can be reduced by decreasing the gap ratio and increasing the gap thickness to a reasonable level. Compared with other numerical simulations and experiment measurement methods, the proposed analytical model can be coded very simply; meanwhile, it intuitively and accurately describes the relationship between the air gap and magnetic characteristics, which is hardly found in the existing technical literature. Although the modeling of transverse magnetic flux in the transition region and simplified configuration of air gaps need further research, the proposed model with explicit mathematical expressions helps to intuitively understand the physical mechanism of an electromagnetic force caused by air gap discontinuities. This understanding is meaningful for noise control, low-noise core design, and condition monitoring for transformers.

## Figures and Tables

**Table 1:** Range of variables and corresponding studied cases.

Variable	Range of Variable	Parameters	Case 1	Case 2	Case 3
$r_{g}$	0.1 to 0.9	w (mm)	2	8	4
		$\mu_{r}$	20,000	2500	1000
w (mm)	2 to 18	$r_{g}$	0.6	0.4	0.8
		$\mu_{r}$	20,000	5000	2500
$r_{g}$	1000, 2500, 5000, 10,000, 20,000	$r_{g}$	0.5	0.6	0.4
		w (mm)	8	6	2

## Data Availability

The data presented in this study are available on request from the corresponding author. The data are not publicly available due to a confidentiality agreement for the project.
